# Interpersonal predictive coding in piano duets: the role of multimodal micro-cues in joint coordination

**DOI:** 10.3389/fpsyg.2026.1808969

**Published:** 2026-08-17

**Authors:** Baoli Zhang, Jiarui Yu, Pengfei Liang

**Affiliations:** 1 School of the Arts, Qingdao University, Qingdao, China; 2 Bangkokthonburi University, Bangkok, Thailand; 3 College of Music, Ludong University, Yantai, Shandong, China

**Keywords:** forward models, joint action, micro cues, piano duet, predictive coding

## Abstract

Piano duets provide an ecologically meaningful model of high-precision joint action. Performers must coordinate note onsets, tempo changes, articulation, and expressive dynamics despite delays in auditory transmission and sensorimotor processing. This temporal constraint suggests that auditory feedback alone cannot fully explain fine-grained interpersonal synchrony. This narrative review examines how pianists may combine prospective prediction with retrospective error correction during joint performance. Drawing on behavioral, computational, motion-capture, and neuroimaging evidence, we propose that performers use learned expectations about a partner’s timing and expressive style together with current auditory, visual, and kinematic information to estimate upcoming musical events. Breathing and body sway may provide phrase-level information about tempo and structural boundaries, although much of this evidence comes from non-piano ensembles. Preparatory hand movements and cueing gestures provide more immediate information about impending note onsets, while auditory feedback remains essential for evaluating expectations and correcting subsequent timing. Computational error-correction and coupled-oscillator models offer complementary descriptions of immediate onset adjustment, longer-term tempo adaptation, and reciprocal temporal influence. Current evidence nevertheless relies heavily on simplified tapping tasks, cross-ensemble extrapolation, and correlational measures of neural coupling. Future research should directly manipulate cue reliability in ecologically valid piano-duet tasks and combine behavioral timing, motion capture, eye tracking, and hyperscanning to test how prediction and feedback jointly support role-dependent interpersonal coordination.

## 1 Introduction

Piano duets provide an ecologically valid model of high-precision joint action. Performers must coordinate note onsets, tempo, and expressive timing while simultaneously controlling their own parts. Networked piano studies show that transmission delays of approximately 10–40 ms can disturb interpersonal timing, whereas adjacent sensorimotor research indicates that auditory error detection and corrective motor responses unfold over a longer processing window (Abalde et al., 2024; Washburn et al., 2021; Weerathunge et al., 2022).

This temporal mismatch suggests that feedback alone cannot explain fine-grained synchrony. Predictive-coding accounts propose that performers combine anticipatory estimates of a partner’s upcoming actions with retrospective information about recently produced events (Abalde et al., 2024; Vuust et al., 2022).

Relevant evidence indicates that performers use auditory, visual, and bodily information differently across stable and uncertain passages, while neural coupling may vary with task demands and interaction structure (D’Amario et al., 2023; Gugnowska et al., 2022; Izen et al., 2023; Lender et al., 2023; Zamm et al., 2021). This review therefore examines how multimodal cues, predictive processing, error correction, and reciprocal adaptation jointly contribute to piano-duet coordination. It further considers how these processes may relate to dynamic role organization and joint action representation (Abalde et al., 2024; Vuust et al., 2022).

## 2 Theoretical framework: from feedback correction to prediction–feedback integration

If temporal coordination in piano duets is explained solely as online error correction based on auditory feedback, it encounters a latency bottleneck. Information about a partner’s note becomes available only after the note has been produced, transmitted, and perceptually processed. Additional time is then required to detect a temporal discrepancy and translate it into a motor adjustment. By the time this adjustment can be implemented, the immediately upcoming note may already be under preparation. In this sense, delay is not merely random noise; it changes the expected temporal relationship between an action and its auditory consequence.

The effect of delay, however, is not uniformly detrimental or beneficial. In a slow dyadic finger-tapping task, Koike et al. (2024) observed a nonlinear pattern: intermediate delays were associated with lower global asynchrony under some conditions, whereas longer delays impaired temporal stability. The improvement observed at intermediate delays was associated with anticipatory responses and reduced dependence on the partner’s immediately preceding interval. However, this task involved simple repetitive tapping rather than expressive musical performance. The finding therefore indicates that participants can reorganize their coordination strategy under a predictable delay; it does not imply that transmission delay generally improves piano-duet performance.

By contrast, transient delayed auditory feedback substantially disrupts the timing and stability of complex sequential piano actions (Yasuhara et al., 2024). This difference may reflect both the greater motor complexity of piano performance and the fact that an unexpected transient delay violates an established action–sound relationship, rather than providing a stable temporal transformation to which participants can adapt. Because Yasuhara et al. (2024) examined individual piano performance rather than interpersonal coordination, their findings demonstrate the sensitivity of sensorimotor control to unexpected auditory consequences but do not by themselves establish mutual prediction. Taken together, these studies support a mixed-control account: prospective prediction helps prepare upcoming actions, whereas retrospective auditory feedback evaluates previous outcomes and updates subsequent predictions.

### 2.1 Predictive coding and Bayesian inference: aligning the future with generative models

Predictive coding describes the brain as a hierarchical generative system in which higher levels generate expectations about upcoming input and lower levels return prediction errors. These errors are precision-weighted: their influence on model updating depends on the estimated reliability of the incoming sensory evidence (Vuust et al., 2022). The Predictive Coding of Music framework emphasizes that music perception and motor control rely on continuous prediction of the next moment’s structure (Vuust et al., 2022). In duet performance, a partner’s sound provides feedback about the event that has just occurred. Performers can use the timing and dynamics of that event to update expectations about the partner’s subsequent notes, tempo trajectory, and phrasing (Vuust et al., 2022). Within a Bayesian account, a prior is an expectation available before the current cue. Repeated rehearsal may teach a pianist, for example, that a particular partner tends to lengthen phrase endings. This gradual acquisition of regularities constitutes statistical learning. The likelihood represents how compatible the currently observed auditory or visual cue is with different possible upcoming events. Combining the learned expectation with the current cue produces an updated, or posterior, estimate of the next onset. This estimate can narrow the range of likely onset times and guide when motor preparation begins (Bottemanne, 2025). When uncertainty increases, cues judged to be more reliable receive greater weight, whereas inconsistent or noisy cues exert less influence on the updated estimate (Bottemanne, 2025; Vuust et al., 2022).

### 2.2 Forward models and self-other integration: from what I will hear to how the partner will play

The core function of forward models is action–outcome prediction. Before an action is executed, the system simulates its likely sensory consequences, enabling faster adjustment and more stable control (Harrison et al., 2023). In joint performance, prediction must also incorporate the partner’s likely action and auditory outcome. Kohler et al. (2023) found that prior rehearsal of a partner’s part was associated with increased activity and connectivity in cortico–cerebellar auditory–motor networks, consistent with the formation of partner-specific action–outcome expectations. In that study, behavioral adaptation referred to the extent to which one performer changed the current inter-keystroke interval in response to the partner’s preceding interval. When subtle asynchronies violated strongly learned partner-specific expectations, cerebellar activity increased while this immediate behavioral adaptation decreased (Kohler et al., 2023). One interpretation is that feedback that conflicted with a strongly learned partner-specific model was treated as less reliable. The pianist may therefore have relied more heavily on the stability of the self-generated timing plan and made less immediate adjustment to the partner. This pattern can be described as a temporary shift toward self–other segregation rather than a complete withdrawal from interpersonal coordination. Because sensory precision was not manipulated directly, this explanation should be treated as an interpretation of the behavioral and neural pattern rather than as a demonstrated precision-weighting mechanism. Self–other integration refers here to incorporating the partner’s timing into one’s own action plan. Self–other segregation refers to maintaining a sufficiently distinct representation of one’s own part and expected timing. A functional balance does not imply equal integration and segregation. Rather, performers must adapt sufficiently to coordinate while avoiding excessive adjustment to unreliable or anomalous partner feedback.

### 2.3 Computational models of temporal error correction

Computational studies of interpersonal timing have used several distinct model families, including linear error-correction models and coupled-oscillator models (Abalde et al., 2024). Linear error-correction models estimate how strongly a performer changes subsequent timing after detecting an asynchrony. Phase correction changes the timing of one or more immediately upcoming onsets without necessarily changing the performer’s longer-term tempo. For example, a pianist who is 30 ms late may slightly shorten the next inter-onset interval to reduce the immediate timing discrepancy. Period correction instead updates the underlying tempo estimate across several events. This may occur when a pianist gradually adopts a slower beat period after repeatedly hearing the partner play later than expected (Abalde et al., 2024).

Coupled-oscillator models use a different mathematical architecture. Each performer is represented as a recurrent timing process, and coupling parameters quantify how strongly the timing state of one performer influences that of the other. “Mutual temporal attraction” therefore refers to the modeled tendency of the two recurrent timing processes to reduce their relative timing difference through reciprocal influence. It does not refer to a neural-network architecture. Plitchenko et al. (2024) fitted a bidirectional delay-coupled oscillator model to joint musical synchronization data. Their model estimated the performers’ intrinsic timing rates, interpersonal coupling strength, and interaction delay. The model was fitted to the partners’ onset-asynchrony time series in two stages. A genetic algorithm first conducted a global search across the permitted parameter ranges, after which a constrained local search refined the estimates. Model fit was evaluated using the root mean square error between the observed and simulated asynchronies, and the best-fitting solution was selected from repeated fits. The full delay-coupled model was also compared with a reduced model in which interpersonal coupling was fixed at zero. The reduced model fitted the observed timing data less accurately, indicating that the coupling parameter contributed information beyond the performers’ intrinsic timing rates. Estimated coupling was stronger in joint performances following normal-feedback solo practice than following delayed-feedback solo practice (Plitchenko et al., 2024).

These parameters describe how condition-related differences in interpersonal adaptation can be represented at the behavioral level. They should not be interpreted as direct neural measures of prediction-error weighting or as separate phase- and period-correction coefficients. From a predictive-coding perspective, the parameters characterize reciprocal adaptation, whereas their relationship to Bayesian prediction-error weighting remains theoretical.

### 2.4 Bidirectional coupling and co-simulation: mutual adaptation rather than unidirectional following

Synchrony does not necessarily involve one performer continuously following the other. Bidirectional coupling means that performer A’s timing influences performer B’s subsequent timing, while performer B’s timing can also influence performer A. The strength of these two directional effects need not be equal. In role-structured drum ensembles, Jacoby et al. (2021) found reciprocal but asymmetric temporal influences, and simulations indicated that the observed role-specific coupling pattern could outperform completely equal coupling or a rigid single-leader organization. This result suggests that asymmetric adaptation can be functional in some ensembles, but it should not be generalized directly to all piano duets. Leader–follower organization may therefore be treated as dynamic rather than permanently assigned. At a phrase entrance, for example, a clearly timed preparatory gesture from one performer may provide a more reliable reference than the partner’s internally maintained beat. The observing performer may temporarily weight that signal more strongly. If subsequent auditory events become inconsistent with the visual cue, the relative influence of the cue may decrease and greater weight may be assigned to auditory or self-generated timing information. This redistribution of influence is compatible with predictive and coupling accounts, but it has not yet been measured directly as a moment-to-moment change in Bayesian precision during piano-duet performance (Abalde et al., 2024; Plitchenko et al., 2024). Co-simulation offers one possible account of how performers represent their own and their partner’s action–sound consequences in parallel. Neuroimaging and EEG hyperscanning studies have reported changes in auditory–motor connectivity and interbrain synchrony during joint musical performance (Kohler et al., 2023; Lender et al., 2023). These findings are compatible with increased interpersonal coordination during demanding passages, but they do not uniquely demonstrate co-simulation. Interbrain synchrony may reflect common sensory stimulation, shared musical structure, similar movements, or higher-level coordination processes, and these alternatives must be distinguished experimentally (Cheng et al., 2024; Müller et al., 2025).

In summary, these accounts operate at complementary explanatory levels. Predictive coding describes how expectations and sensory evidence may be combined. Forward models describe anticipated action–outcome relationships. Error-correction and coupled-oscillator models quantify observable temporal adjustments between performers. Neuroimaging and hyperscanning studies examine the neural processes associated with self–other coordination. Together, these approaches support a multilevel account of joint music making, although they do not yet constitute a single empirically validated computational architecture (Abalde et al., 2024).

## 3 Multimodal micro-cues as predictive evidence

From a predictive coding perspective, duet synchrony combines past acoustic outcomes with earlier multimodal micro-cues, including breathing, trunk and head sway, pre-keystroke preparation, and subtle timing and dynamic deviations in the sound stream. These cues provide current sensory evidence that can update prior expectations and reduce uncertainty about upcoming events such as onsets, tempo changes, and accents (Abalde et al., 2024). Micro-cues are not mechanisms in themselves. They are information sources that can enter a predictive process. A performer detects the cue, relates it to previously learned cue–outcome associations, narrows the estimated onset window, prepares an action, and then uses the resulting sound to confirm or update that estimate. Existing piano-duet studies support specific components of this proposed sequence rather than the entire mechanism. Preparatory gesture kinematics have been shown to influence synchronization success, and performers increase visual monitoring during temporally uncertain passages (Bishop and Goebl, 2018; Bishop et al., 2019). These findings support the cue-extraction and anticipatory-preparation components of the account. However, no existing piano-duet study has directly demonstrated the complete causal sequence from micro-cue perception to Bayesian prediction-error minimization and enhanced joint agency. The latter links therefore remain hypotheses for future testing.

### 3.1 Pre-sound preparatory cues: breathing and sway as low-frequency carriers

#### 3.1.1 Breathing: from a physiological act to a temporal anchor

In ensemble settings, breathing is often structured as a readable timing signal rather than merely an energy source. Choir studies show increased respiratory phase synchrony during performance, with conductors’ breathing changes often preceding singers’, consistent with a directional, low-frequency carrier of tempo and phrase boundaries (Delius and Müller, 2023; Lange et al., 2022). Preparatory gestures can shape inhalation and subsequent vocal output, linking breath to onset timing (Platte et al., 2024), and expert performers co-regulate breathing with musical structure and anticipatory planning (Cara and Mitrovic, 2024). By extension, an audible or visible in-breath before an entrance may serve as a temporal cue in piano duets by signaling an imminent event. However, this possibility has not yet been tested directly in piano-duet experiments.

#### 3.1.2 Preparatory body sway: visualizing global tempo

Like breathing, body sway conveys longer-timescale structure without requiring beat-by-beat alignment, expressing global tempo, tension trajectories, and phrase segmentation through continuous motion. Musical texture refers to the organization and relative prominence of simultaneously sounding parts. In a homophonic texture, one part carries a clearly identifiable melody while the remaining parts provide accompaniment, producing a comparatively explicit role hierarchy. In a polyphonic texture, several parts possess greater melodic independence and the hierarchy is less pronounced. Video-based computational analyses show that directional influence in ensemble sway varies with musical texture, with more hierarchical textures producing clearer leader–follower patterns and coupling strength changing across phrase positions (Sabharwal et al., 2024). From a predictive perspective, body sway may provide phrase-level evidence about tempo, role organization, and impending structural change. However, Sabharwal et al. (2024) measured movement directionality rather than performers’ prior expectations or keystroke-level predictions directly.

### 3.2 Kinematic signatures and visual capture: communicative pre-keystroke movements as high-frequency information

#### 3.2.1 Pre-keystroke hand lift and cueing actions: clearer kinematics for lower uncertainty

In duets, the most predictive visual cues are often not the keystroke that is unfolding. They are kinematic changes before the keystroke, including hand lifting, rising acceleration, movement peaks, and return trajectories. Motion-capture work on starting cue gestures in piano duets shows that ensemble synchrony does not improve simply because performers can see each other. It depends on the kinematic structure of the cueing movement. For example, synchrony is better when cue gestures show clearer acceleration features at critical moments, such as stable mapping between acceleration peaks and troughs and the intended onset. Synchronization success is jointly shaped by the interpretability and conventionality of the gesture and its kinematic properties (Bishop and Goebl, 2018). These findings can be interpreted within a predictive-coding framework. More discriminable lift height, velocity, and acceleration patterns may allow the observing performer to estimate a narrower range of possible onset times. However, Bishop and Goebl (2018) measured synchronization and gesture kinematics rather than Bayesian posterior distributions directly.

#### 3.2.2 Peripheral vision and intermittent glances: context-dependent visual monitoring

Pianists cannot, and do not need to, fixate continuously on the partner. Evidence indicates that reliance on visual cues is context dependent. When musical structure makes prediction more difficult, such as after long rests or during tempo-change passages, performers use visual cues more effectively. Even when the partner’s audio input is absent, synchrony after a long rest can be partially maintained using visual cues alone (Bishop and Goebl, 2015). Mobile eye-tracking work further shows that during temporally unstable passages, performers markedly increase visual monitoring of the partner, whereas during rhythmically stable passages, visual interaction decreases (Bishop et al., 2019). Together, these findings support a resource-allocation interpretation. During rhythmically stable passages, performers can rely more heavily on internal timing models and peripheral information. During uncertain passages, such as re-entries after rests or tempo changes, brief visual monitoring may provide additional information for recalibrating onset expectations (Bishop and Goebl, 2015; Bishop et al., 2019).

#### 3.2.3 From movement for production to movement for communication: the core function of communicative kinematics

Taken together, these findings support a stronger functional account. Many pre-keystroke movements are not solely for producing sound. They also have communicative value. By exaggerating or structuring kinematic features, performers make their temporal intentions easier for a partner to decode and thereby reduce predictive uncertainty. The functional value of a kinematic cue therefore depends on whether its timing and form allow the observer to distinguish among possible upcoming actions. This principle also has implications for human–robot musical interaction, in which artificial partners must both recognize and generate readable gestures marking starts, stops, and beats (Gao et al., 2024).

### 3.3 Auditory feedback and temporal correction

Prediction does not replace auditory feedback. Auditory input both provides essential partner information and enables performers to evaluate and update predictions. In piano duets, coordination is strongest when partner audio is available, drops sharply when partner audio is removed and only vision remains, and is less affected by removing self feedback, consistent with partner audio serving as evidence for testing priors (Bishop and Goebl, 2015). When temporal errors occur, phase correction refers to adjusting one or more upcoming onset times to reduce the immediate relative timing discrepancy. Period correction refers to a slower modification of the underlying tempo estimate across several events. In a coupled-oscillator account, “re-locking” describes the reduction of the relative phase difference between two recurrent timing processes until corresponding events again occur near the intended temporal relationship. Plitchenko et al. (2024) modeled joint synchronization using a bidirectional delay-coupled oscillator rather than a separate linear phase–period correction architecture. Their findings therefore support the importance of reciprocal coupling and interaction delay, but they should not be described as demonstrating that skilled duos universally prioritize phase correction over linear correction.

## 4 Shared goals, role-dependent coordination, and joint agency

The preceding sections describe processes that may support temporal coordination, including anticipatory cues, auditory feedback, and reciprocal error correction. These processes should be distinguished from the performers’ shared goal and subjective sense of joint agency. A shared goal is normally established before the duet begins, whereas the strength of joint agency—the experience that an outcome has been produced together—may vary during performance. Coordination mechanisms can also support different interaction organizations depending on musical structure. Unison passages may encourage relatively symmetrical adaptation, melody–accompaniment textures may produce asymmetric influence, and turn-taking or improvisatory passages may involve alternating control (Abalde et al., 2024).

### 4.1 Dynamic leadership: Cue reliability and the allocation of control

In Bayesian terms, precision is the inverse of uncertainty, commonly formalized as inverse variance. Precision weighting determines how strongly a prediction error or sensory cue influences belief updating and motor adjustment. It therefore refers to estimated informational reliability rather than a performer’s subjective self-confidence (Limanowski et al., 2024). In a duet, a clearly timed preparatory gesture may temporarily provide a more precise estimate of an upcoming onset than an ambiguous auditory or visual cue. The partner may therefore weight that signal more strongly when preparing the next action. As texture or cue reliability changes, the direction of influence may also change. Movement-based directionality varies with musical texture and phrase position, providing behavioral evidence that interpersonal influence is not fixed (Sabharwal et al., 2024). Hyperscanning research has also identified different patterns of interpersonal neural coupling in symmetrical and melody–accompaniment conditions (Yuan et al., 2025). These findings demonstrate role-dependent neural organization, but they do not directly measure Bayesian precision or prove that the melodic performer is the causal leader. Evidence from nonmusical haptic joint action further shows that movement timing and force can convey implicit confidence and influence control allocation (Pezzulo et al., 2021), although its applicability to piano duets remains to be tested.

### 4.2 Joint agency as a possible correlate of successful coordination

Performers normally begin a duet with an explicit shared goal, such as producing a coherent interpretation of the same musical work. This pre-existing shared goal should be distinguished from the sense of joint agency that may arise during performance. Joint agency refers to the subjective experience that the performers are jointly controlling an action and its outcome, rather than each performer acting independently. General joint-action literature describes a we-mode orientation in which participants interpret their actions in relation to shared goals, collective responsibility, and coordinated outcomes (Sarabi et al., 2025). However, the presence of a shared goal does not necessarily mean that performers experience themselves as a single or merged agent.

Empirical evidence indicates that joint agency is influenced by the organization and quality of interpersonal coordination. In a cooperative tone-sequence task, Bolt et al. (2016) found that participants reported stronger joint agency when both partners adapted to one another than when coordination was primarily unidirectional. Better temporal coordination was also associated with stronger feelings of shared control. Partner predictability provides another relevant factor. Bolt and Loehr (2017) found that participants experienced stronger joint agency when coordinating with a temporally predictable partner, even after accounting for their own timing accuracy and variability. These findings suggest that mutual adaptation and the ability to anticipate a partner’s actions can contribute to joint agency, although the tasks involved relatively simple tone sequences rather than ecologically complete musical performances.

More direct musical evidence comes from Zhou et al. (2023), who compared synchronized constant-pitch sequences with musical duets containing richer metrical and harmonic relations between the partners’ parts. Participants reported stronger joint agency during the musical duets than during the constant-pitch sequences, even when the two conditions were matched for synchronization quality. Post-performance interviews further indicated that joint agency was shaped by participants’ musical knowledge and by their perceptions of performance quality, task difficulty, and enjoyment. Thus, accurate synchrony alone is insufficient to explain the experience of acting together. The musical relationship between the performers’ parts and the performers’ interpretation of the joint outcome also contribute to joint agency.

Within the present predictive-coding framework, successful prediction and stable reciprocal coordination are therefore proposed as possible contributors to joint agency. Predictable partner actions may reduce uncertainty and facilitate the integration of the partner’s timing into one’s own action plan. Nevertheless, current evidence does not establish a direct causal sequence in which reduced prediction error automatically shifts control from a self-centered representation to a fully system-centered or merged representation. Joint agency is better treated as a graded and context-dependent subjective correlate of coordinated performance. Future piano-duet studies should measure prediction error, partner predictability, synchronization quality, and subjective joint agency within the same task to test this proposed relationship directly.

### 4.3 Co-representation of individual and joint outcomes

Co-representation refers to encoding not only one’s own action and sensory outcome but also the partner’s contribution and the joint result. Marschner et al. (2024) examined this behaviorally by testing whether performance was influenced by previously learned individual-level or joint-level action–outcome relationships. Sensitivity to the joint-level mapping suggests that participants represented the dyadic outcome rather than encoding only their own contribution. This result supports joint-level representation, although it does not imply that the two individual representations become fully merged.

Complementary evidence comes from nonmusical joint statistical learning. Zheng and Wang (2024) reported behavioral learning and interference effects accompanied by changes in theta-band brain-to-brain coupling. These findings indicate that self–other integration can vary during joint learning. Because the task was not musical and interbrain coupling may also reflect common stimulation or shared task structure, the result should be treated as indirect evidence rather than direct proof of a unified piano-duet sound representation. Shared intentionality should not be treated solely as a product that emerges after successful synchronization. The performers generally enter the duet with a shared performance goal. During performance, mutual prediction and reciprocal adaptation may strengthen the representation of joint outcomes and the subjective sense of joint agency. Dynamic leadership describes momentary changes in whose information exerts greater influence. Shared goals, temporal coordination, co-representation, and joint agency should therefore be distinguished as related but nonidentical levels of joint action.

### 4.4 Limitations and boundary conditions of current evidence

The predictive-coding account developed in this review is supported by converging findings, but the current evidence remains heterogeneous in task, performance context, and level of explanation. A first limitation concerns ecological validity. Several influential studies have used repetitive finger-tapping or simplified tone-sequence tasks because these paradigms allow precise manipulation of delay, partner predictability, and temporal error. For example, Koike et al. (2024) demonstrated that dyadic timing can change nonlinearly across different delay conditions. Such tasks are valuable for isolating basic coordination processes, but they do not reproduce the full demands of piano-duet performance, which requires simultaneous control of pitch, fingering, articulation, dynamics, phrasing, score reading, and role-specific musical interpretation. Findings from simplified synchronization tasks should therefore be treated as evidence for general temporal-control principles rather than as complete models of expert piano interaction. As emphasized by Abalde et al. (2024), ecologically valid joint music making involves interacting behavioral, cognitive, neural, and social processes that cannot be fully captured by a single reduced task.

A second limitation concerns the transfer of evidence across musical settings. The proposal that breathing may provide anticipatory information in piano duets is based largely on evidence from choir singing and wind-instrument performance. Respiratory synchronization and conductor–singer directionality have been observed during ensemble singing (Delius and Müller, 2023), while expert flute performance demonstrates close coupling between breathing, anticipation, musical structure, and practice (Cara and Mitrovic, 2024). These findings make it plausible that visible or audible inhalation could signal phrase entrances in a piano duet, but this function has not yet been tested directly in pianists. A similar limitation applies to body sway. Sabharwal et al. (2024) showed that movement directionality varies with musical texture, phrase position, and role organization in concert-performance videos. However, these results were obtained from ensemble contexts that differ from the seating arrangement, visual access, bodily constraints, and hand-centered coordination demands of piano duets. Breathing and sway should therefore be described as candidate information sources whose functions in piano performance require direct experimental confirmation.

A third limitation concerns the interpretation of interbrain synchrony. Hyperscanning studies have reported changes in neural coupling during joint musical performance, but interpersonal neural synchrony is not a process-pure measure of shared prediction or shared intentionality. Two performers may exhibit similar neural activity because they hear the same rhythm, follow the same score, execute comparable movements, or respond to the same external event. Neural coupling may also reflect common attentional demands rather than direct information exchange between performers. Reviews of musical hyperscanning therefore caution that endogenous interpersonal coordination must be distinguished from synchrony produced by common sensory stimulation, shared task structure, and parallel motor behavior (Cheng et al., 2024; Müller et al., 2025). Stronger interbrain synchrony should consequently not be interpreted automatically as evidence that two performers have formed a unified representation or entered a we-mode. More convincing evidence would require experimental designs that control common input, manipulate directional access to partner cues, and relate neural coupling to independently measured changes in behavioral adaptation and subjective joint agency.

A fourth limitation is that several central constructs in the proposed framework have rarely been measured directly. Bayesian precision is often inferred from changes in cue use, adaptation strength, or neural activity rather than estimated as an explicit trial-by-trial variable. Likewise, joint agency is commonly assessed after performance through retrospective ratings, whereas the proposed changes in prediction error and cue weighting occur continuously. Current studies therefore do not establish a direct causal sequence in which reduced uncertainty leads to stronger precision weighting, improved synchrony, and an enhanced sense of joint agency. These relations remain theoretically plausible but empirically underdetermined. Future studies should manipulate the reliability of auditory and visual cues and collect synchronized measures of onset error, adaptation, gaze, movement, neural activity, and subjective control. This would make it possible to test whether changes in cue precision predict both objective coordination and the performers’ experience of acting together.

Finally, predictive coding, linear error-correction models, and coupled-oscillator models operate at different explanatory levels and should not be treated as a single unified architecture. Predictive coding provides a general theoretical account of how expectations and sensory evidence may be integrated. Linear phase- and period-correction models quantify how performers adjust immediate onset timing and longer-term tempo after detecting temporal discrepancies. Coupled-oscillator models describe reciprocal influence between recurrent timing processes. These models can offer complementary explanations of the same performance, but their parameters are not interchangeable. A coupling coefficient is not a direct measure of Bayesian precision, and a phase-correction coefficient is not itself a neural prediction error. At present, the relationship among these levels remains interpretive rather than formally established (Abalde et al., 2024). Progress will require models that generate testable cross-level predictions while preserving the distinction between theoretical constructs, behavioral parameters, and neural measurements.

## 5 Conclusion and future directions

Piano-duet synchrony depends on an interaction between prospective prediction and retrospective feedback. Breathing and body sway may provide phrase-level information, although direct piano-duet evidence remains limited. Preparatory kinematics can provide more immediate information about upcoming onset timing and dynamics. Auditory feedback remains essential for evaluating these expectations and supporting subsequent temporal correction. Current evidence is limited by reliance on simple tapping tasks, evidence transferred from non-piano ensembles, and correlational measures of interpersonal neural coupling. Predictive precision, shared intentionality, and co-representation are rarely measured directly within the same experiment. Tactile and vibratory signals, covert intention markers, and differences across musical styles also remain underexamined. Future work should combine behavioral timing, motion capture, eye tracking, and EEG hyperscanning in ecologically valid duet tasks. Cross-style comparisons could test whether coordination differs between classical and improvisatory performance. Human–AI co-performance designs could further examine whether artificial partners that recognize and generate readable preparatory movements improve coordination under uncertainty.
